# The effect of trauma care systems on the mortality of injured adult patients: A protocol for systematic review and meta-analysis

**DOI:** 10.1097/MD.0000000000022279

**Published:** 2020-09-25

**Authors:** Wu Jifang, Yang Liping, Zhu Jing, Song Jie

**Affiliations:** Trauma Center, The First People's Hospital of Lianyungang, Lianyungang, Jiangsu Province, China.

**Keywords:** meta-analysis, mortality, trauma care systems

## Abstract

**Purpose:**

The aim of this study was to have a comprehensive evaluation of the effect of trauma care systems on the mortality of injured adult patients.

**Materials and methods:**

This protocol established in this study has been reported following the Preferred Reporting Items for Systematic Review and Meta-Analysis Protocols. Web of Science, PubMed, EMBASE, Scopus, and the Cochrane Library were searched for all clinical trials evaluating the effect of trauma care systems on the mortality of injured adult patients until July 31, 2020. We will use a combination of Medical Subject Heading and free-text terms with various synonyms to search based on the eligibility criteria. Two investigators independently reviewed the included studies and extracted relevant data. The odds ratio (OR) and 95% confidence intervals (CIs) were used as effect estimate. I-square (I^2^) test, substantial heterogeneity, sensitivity analysis, and publication bias assessment will be performed accordingly. Stata 15.0 and Review Manger 5.3 are used for meta-analysis and systematic review.

**Results:**

The results will be published in a peer-reviewed journal.

**Conclusion:**

The results of this review will be widely disseminated through peer-reviewed publications and conference presentations. This evidence may also provide a comprehensive evaluation of the effect of trauma care systems on the mortality of injured adult patients.

**Registration number:**

INPLASY202080058

## 1 Introduction

Trauma is one of the leading causes of death worldwide.^[1]^ Approximately 5.8 million people die each year as a result of trauma.^[2]^ Before the modern era of resuscitative medicine and surgery, and the reorganization of emergency hospital care into major trauma centers, the likelihood of survival following a traumatic injury was down to the individual's physiological response to injury.^[3,4]^ Survival is achieved via a complex set of metabolic, endocrine, and immunological pathways that mobilize fuel sources and minimize blood loss, so that our vital organs may continue to be perfused and function.^[5]^

Implementation of a comprehensive mature trauma care system has led to a decrease in mortality and morbidity among injured patients in many high-income countries such as the United States (US), Canada, and Australia.^[6]^ The trauma care system is designed to provide specialized trauma care for all injured patients and to enhance accessibility to acute health care facilities among those injured, ensuring they have access to higher levels of care.^[7]^ A coordinated trauma care system encompasses authority, strategies, and services to optimize injury prevention, prehospital emergency medical service (EMS), acute care hospitalization, and posthospital care.^[1]^ Currently, there is a growing interest in how the trauma care systems could affect this physiological response to major trauma and how to manipulate recovery to improve patient outcomes further.^[1]^ However, there is rare systematic review and meta-analysis to access the effect of trauma care systems on the mortality of injured adult patients.

To tackle with those problems, we further performed a systematic review and meta-analysis to provide a comprehensive evaluation of the effect of trauma care systems on the mortality of injured adult patients.

## 2 Study aim

The aim of our study is to have a comprehensive evaluation of the effect of trauma care systems on the mortality of injured adult patients.

## 3 Methods

The protocol of our meta-analysis followed the guideline of the Preferred Reporting Items for Systematic Review and Meta-Analysis Protocols (PRISMA-P) recommendations.^[8]^ It has been registered with INPLASY database as INPLASY202080058 (https://inplasy.com/inplasy-2020-8-0058/).

### 3.1 Search strategy

A systematic search was performed in Web of Science, PubMed, EMBASE, Scopus, and the Cochrane Library until July 31, 2020. The MeSH search and text word will be used with the terms related to “trauma care system.” To perform a comprehensive and focused search, experienced systematic review researchers will be invited to develop a search strategy. An example of search strategy for PubMed database is summarized in Table 1, which will be modified and used for the other databases. The reference lists of all relevant studies will be searched for additional relevant studies not retrieved from the electronic database search.

**Table 1:**
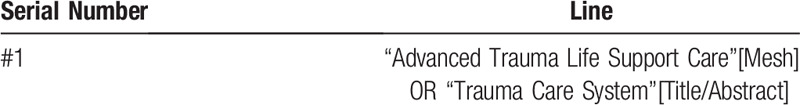
Searching strategy in PubMed.

### 3.2 Eligibility criteria

Inclusion criteria include English language, randomized controlled trials, nonrandomized controlled trials, before and after studies, interrupted time series, cohort studies, and case–control studies. Studies evaluating the effect of a trauma system on the primary outcome, adult patient mortality, will be included. Due to the numerous definitions of a trauma system, studies will be deemed eligible for inclusion if the study authors define their intervention as a trauma system and if the intervention has 2 of the following clinical components identified by the Trauma Association of Canada.^[9]^

Exclusion criteria include studies solely reporting pediatric outcomes and studies without a comparator will be excluded. Combat data and trauma systems in developing countries will also be excluded. The Central Intelligence Agency World Factbook will be used to identify developed and developing countries.

### 3.3 Study selection

**Figure 1:**
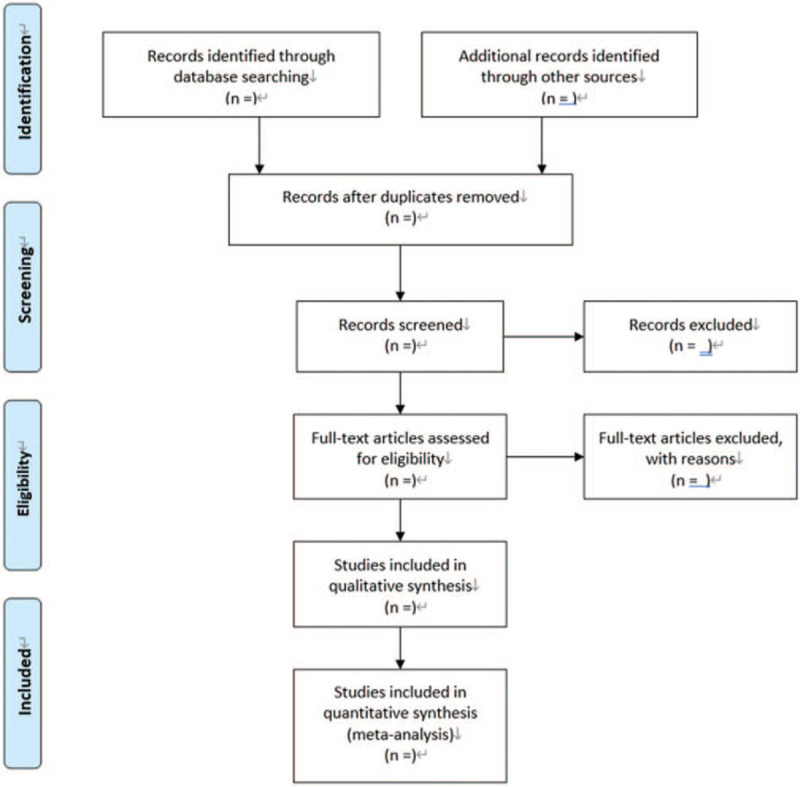
Flow diagram: selection process for the studies.

All initial records from 4 electronic databases will be imported into the web-based systematic review Rayyan software.^[10]^ First, the titles and abstracts of records will be reviewed independently by 2 reviewers to identify potential trials according to eligibility criteria. Then, full-text of all potentially relevant trials will be downloaded to make sure eligible trials. Any conflict will be resolved by discussion. A flow diagram (Fig. 1) will be used to describe the selection process of eligible papers.

### 3.4 Data extraction and management

The data will be extracted out by 2 independent reviewers in accordance with the Cochrane Handbook of Systematic Reviews of Interventions. Two investigators will independently screen all the included studies. The main outcome will be the mortality of injured adult patients. Additional outcomes will be the disability rate and length of hospital stay.

### 3.5 Risk of bias of individual study and quality assessment

Two reviewers will evaluate independently the risk of bias of included studies using a modified Version of Cochrane tool^[11]^ in which we will check for allocation concealment, blinding, incomplete outcome data, selective reporting, and other bias, each of which makes high risk, low-risk, and unclear grades. Any discrepancy was resolved by discussion or by a third reviewer.

### 3.6 Data analyses

All the statistical analysis was achieved in Rev Man 5.3 (Cochrane Library Software, Oxford, UK). The trial data were processed according to the Cochrane Reviewers’ Handbook. We calculated standard deviations (SDs) based on 95% confidence interval (CI) or *P* values if not reported. Dichotomous data were expressed as odds ratio (OR), while continuous variables were presented as mean difference (MD), both with 95% CI. The *z* test was performed to determine all pooled effects, and statistical significance was defined as *P* < .05. If *I*^2^ < 50% or *P* > .1 was reported according to the Chi-square-based Q test and *I*^2^ test, heterogeneity was assessed as low, and the fixed-effects model was used. Otherwise, the random-effects model was used. Certain literature was removed each time for sensitivity analysis.

### 3.7 Publication bias

If included studies were more than 10, funnel plot will be used to identify the possible publication bias. In addition, Egg regression and Begg tests will be utilized to detect the funnel plot asymmetry.^[12]^

### 3.8 Subgroup analysis

If there is enough research, we will conduct a subgroup analysis to investigate differences in age, gender, and so on.

## 4 Discussion

Previous studies have reported that trauma care systems could have effects on the mortality of injured adult patients.^[13,14]^ Thus, this systematic review and meta-analysis will have a comprehensive evaluation of the effect of trauma care systems on the mortality of injured adult patients. The results of this review will be widely disseminated through peer-reviewed publications and conference presentations. This evidence may also assist clinicians in assessing the effect of trauma care systems on the mortality of injured adult patients.

## Author contributions

Conceptualization: Wu Jifang and Song Jie; Acquisition: Wu Jifang, Yang Liping, Zhu Jing and Song Jie; Registration: Song Jie; Methodology: Wu Jifang, Yang Liping, Zhu Jing and Song Jie; Project administration: Wu Jifang and Song Jie; Writing and original draft: Wu Jifang, Yang Liping, Zhu Jing and Song Jie.

**Conceptualization:** Wu Jifang, Song Jie.

**Formal analysis:** Wu Jifang.

**Funding acquisition:** Song Jie.

**Investigation:** Wu Jifang, Yang Liping, Song Jie.

**Methodology:** Wu Jifang, Song Jie.

**Project administration:** Yang Liping.

**Resources:** Wu Jifang, Zhu Jing, Song Jie.

**Software:** Zhu Jing, Song Jie.

**Supervision:** Yang Liping, Song Jie.

**Validation:** Zhu Jing.

**Writing – original draft:** Song Jie.

**Writing – review & editing:** Song Jie.
